# Decision-making in interhospital transfer of traumatic brain injury patients: exploring the perspectives of surgeons at general hospitals and neurosurgeons at neurotrauma centres

**DOI:** 10.1186/s12913-024-11968-z

**Published:** 2025-02-11

**Authors:** Mathias Cuevas-Østrem, Torben Wisborg, Olav Røise, Eirik Helseth, Elisabeth Jeppesen

**Affiliations:** 1 Faculty of Health Sciences, University of Stavanger, Stavanger, Norway; 2 Department of Research, Norwegian Air Ambulance Foundation, Oslo, Norway; 3 Norwegian Trauma Registry, Division of Orthopaedic Surgery, Oslo University Hospital, Oslo, Norway; 4 INTEREST: Interprofessional Rural Research Team-Finnmark, Faculty of Health Sciences, University of Tromsø - the Arctic University of Norway, Hammerfest, Norway; 5 Norwegian National Advisory Unit On Trauma, Division of Emergencies and Critical Care, Oslo University Hospital, Oslo, Norway; 6 Department of Anaesthesiology and Intensive Care, Hammerfest Hospital, Finnmark Health Trust, Hammerfest, Norway; 7 Institute of Clinical Medicine, Faculty of Medicine, University of Oslo, Oslo, Norway; 8 Department of Neurosurgery, Oslo University Hospital, Oslo, Norway; 9 Faculty of Health Sciences, VID Specialized University, Oslo, Norway

**Keywords:** Traumatic Brain Injury, Interhospital, Transfer, Elderly, Old, Geriatric, Frailty, Comorbidity, Functional impairment, Qualitative study

## Abstract

**Background:**

Traumatic brain injury (TBI) is a significant public health concern. Advancing age and comorbidities are associated with a reduced probability of being transferred to neurotrauma centres (NTCs) from non-neurosurgical acute care trauma hospitals (ACTHs). However, the extent to which these decisions reflect well-considered treatment-limiting decisions and which influence other factors have on the decision-making process remains unclear.

**Objective:**

To increase the understanding of adults’ access to NTC care by exploring the decision-making process for interhospital transfer of patients with isolated TBI, elucidating factors influencing these decisions.

**Methods:**

Fifteen surgeons and neurosurgeons from four hospitals in Norway were recruited through purposive sampling to four semi-structured focus group interviews. Surgeons represented ACTHs and neurosurgeons NTCs, and all participants were responsible for TBI patients' initial care and transfer decisions. Interviews were thematically analysed.

**Results:**

We identified several factors influencing transfer decisions, captured in six main themes under one overarching theme; ‘The chance of a favourable outcome’. The six main themes reflect surgeons’ and neurosurgeons’ decision-making process, which included clinical and system-level factors: (A) ‘Establish TBI severity: Glasgow Coma Scale score and head CT’, (B) ‘Preinjury health status: comorbidity, functioning, and age’, (C) ‘Distance from ACTH to NTC: distance is time and time is brain’, (D) ‘Uncertainty and insecurity’, (E) ‘Capacity at NTC’, and (F) ‘Next of kin involvement’.

**Conclusion:**

On-call surgeons and neurosurgeons responsible for making transfer decisions for TBI patients emphasise the importance of patient-centred decisions, including individual patients’ risk factors and overall health status.

**Supplementary Information:**

The online version contains supplementary material available at 10.1186/s12913-024-11968-z.

## Introduction

Traumatic brain injury (TBI) is a significant public health concern, causing nearly 2 million hospital admissions in Europe annually, most of them in older adults [1–3]. As much as 40–50% of all patients with moderate-to-severe TBI are primarily admitted to non-neurosurgical acute care trauma hospitals (ACTHs), making interhospital communication and transfer to neurotrauma centres (NTCs) essential for patients who require neurosurgery or neurocritical care [4–7]. The risk of primary admission to ACTHs and subsequent non-transfer to NTCs increases with advancing age [4, 5, 8, 9]. These findings raise important questions about the decision-making process regarding access to specialised neurotrauma care for older TBI patients.

Guidelines recommend involving NTCs early in decisions about TBI care when appropriate, typically via a telephone consultation between the responsible surgeons on-call at the ACTH and the NTC [10, 11]. Information about injury type and severity, as indicated by head computed tomography (CT) findings, neurological deficits, and Glasgow Coma Scale (GCS) scores, are decisive determinants for transfer decisions because they define indications for emergency neurosurgery or neurointensive care [10, 12, 13]. However, literature and guidelines are scarce on how other factors influence transfer decisions, although evidently, some patients remain at ACTHs despite moderate-to-severe TBIs [4–6]. Advanced age and comorbidities have been associated with a reduced transfer probability, most likely due to their associations with poor outcomes [4, 14–16]. The potential influence other factors have on the transfer decision and how clinicians weigh them against each other remains to be explored. With the increasing number of older TBI patients, it is pertinent to understand whether the reduced transfer rates and treatment intensities found in previous studies represent well-considered treatment-limiting decisions or are the results of self-fulfilling prophecies about poor outcomes [13, 16, 17].

This study aimed to explore the decision-making process for interhospital transfer of patients with isolated TBI from ACTHs to NTCs, elucidating factors influencing these decisions. Information from this study will increase the understanding of what determines transfer to specialised neurotrauma care for patients primarily admitted to ACTHs and how complex information is collected and applied under time pressure. Also, factors that have not been considered to influence transfer decisions in previous studies may be identified, which can inform future studies and practice. Together, this would increase transparency for all stakeholders, including healthcare professionals in prehospital and in-hospital settings who frequently encounter these patients.

## Methods

### Study design

We conducted a focus group study using thematic analysis to explore the factors influencing transfer decisions inductively [18]. The study was conducted in line with a pre-published protocol and is reported in line with the consolidated criteria for reporting qualitative research (COREQ) [19, 20] (Appendix 1).

### Setting

**Fig. 1:**
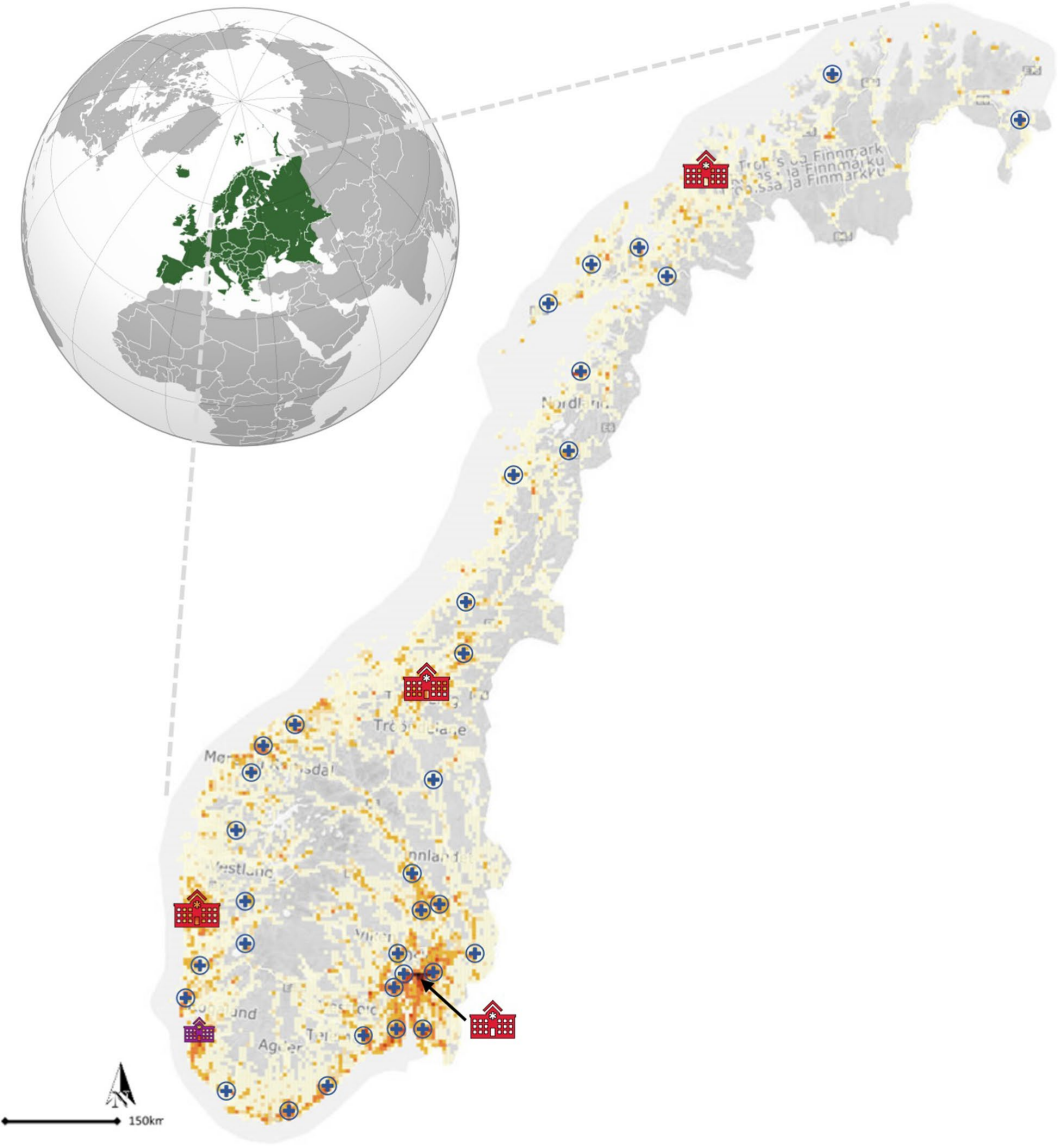
Map of Norway showing population density and locations of neurotrauma centres and acute care trauma hospitals. Grey to orange colours on the map represent increasing population density. Neurotrauma centres are shown in red (regional trauma centres) and violet (one acute care trauma hospital with neurotrauma services). Acute care trauma hospitals are shown in blue. Left: Norway’s location in Europe. Modified and reused from [4] under CC BY 4.0 license. The figure is built on public domain content from Wikimedia Commons and Statistics Norway

Norway, with a population of approximately 5.4 million, has a diverse population density and often considerable distances between hospitals (Fig. 1). The country’s trauma care is organised within the national Norwegian trauma system, which comprises four regional trauma systems and a well-developed prehospital system [11]. Each regional system follows regional health trust borders and includes multiple ACTHs and one designated trauma centre (TC). The TCs are teaching hospitals with all medical specialities available and serve as the referral hospital in the region [11]. Neurosurgical and neurocritical care services are available 24/7 at all four TCs and one ACTH (Stavanger University Hospital), jointly called NTCs in this study. All hospitals in the Norwegian trauma system have 24/7 trauma team availability, emergency general surgery, and critical care and high-dependency units, and teleradiology enabling digital transfer of CT images between hospitals. Neurosurgical interventions are only performed at NTCs [21]. Patients with suspected severe TBI should bypass the nearest hospital and be directly transported to an NTC, if available within 45 min (Appendix 2) [11]. Anaesthesiologist-led air or ground emergency medical services are available throughout the country for advanced prehospital care and interhospital transfers.

### Participants

We recruited participants through purposive sampling from hospitals within two regional health trusts to facilitate information sampling from areas with potentially different views and experiences. Within each health trust, one ACTH and one NTC were chosen to get information from cooperating hospitals, concluding with four interviews from four hospitals. The two ACTHs we recruited participants from were small-to-medium-sized, one admitting 200–400 trauma patients/year and the other < 200 [22]. The transfer distance to their corresponding NTC was < 100 km with a drive time for regular road traffic of 30–90 min for one hospital and > 300 km with a drive time of > 240 min for the other (www.openstreetmap.org/copyright – OpenStreetMap Foundation, Cambridge, United Kingdom).

Participants were eligible if they were A) surgical residents or attendings at ACTHs with more than one year of experience as trauma team leaders, responsible for on-call consultations with their corresponding NTC, or B) neurosurgical residents or attendings at NTCs responsible for answering on-call consultations from ACTHs about interhospital transfer and subsequent neurosurgical management. Invitations were sent to key personnel in each hospital, who forwarded the invitation to all potential candidates in their departments.

### Interview guide and data collection

The interview guide development process was informed by current literature and experts in neurosurgery, general and orthopaedic surgery, anaesthesiology, trauma system development, and qualitative methods. Discussions were held in one physical meeting and during revisions of the interview guide via e-mail. The interview guide covered topics such as current on-call consultation practices and which factors were considered to make a transfer decision, and questions were not restricted to focus on older patients to allow open exploration of the role of age and age-related features (Appendix 3). Questions were framed on isolated TBI because various factors' influence may differ in cases with significant extracranial injuries. The interview guide was pilot tested on one registrar in orthopaedic surgery who met eligibility criteria and worked at a nonparticipating hospital. Subsequently, some questions were clarified, and new ones were added.

Data were collected through a total of four qualitative semi-structured focus group interviews conducted during the COVID-19 pandemic between April 2020 and June 2021, with 3–5 participants in each. One focus group interview was performed via video communication, while the remaining were in-person. The same two researchers conducted all interviews to ensure intimate knowledge of the material: one was a PhD student on the topic of patient safety in trauma care and an internal medicine resident who had received training in qualitative research (MCØ), and the other was a senior trauma system researcher with extensive experience as an intensive care unit (ICU) nurse in a remotely located hospital (EJ). Both entered the project with first-hand experience with interhospital transfer of TBI patients, and reflexivity concerning this was explicitly discussed throughout the planning and conducting of this study.

Each interview lasted approximately one hour and was audio-recorded and transcribed verbatim. The interviews began with a statement that the goal was to gain increased knowledge about the processes behind interhospital transfer of traumatic brain injury patients and not to evaluate their current practice. The interviews were conducted during regular working hours at the hospitals where participants worked. After each interview, the interviewers debriefed and made field notes that were used to inform the analysis. After the fourth preplanned focus group interview, we discussed saturation (MCØ and EJ). We decided to conclude with data sampling because the groups had expressed perspectives with a large degree of overlap, and rich information about factors influencing transfer decisions had been obtained.

### Analysis

We analysed data using Thematic Analysis in an iterative process involving multiple steps [18]. First, the transcripts were read and re-read to become familiar with the data. Second, all transcripts were coded inductively by one author (MCØ) who created an initial coding structure. We aimed to code the data as given by participants in the interviews, rather than by pre-selected theories or topics. Third, candidate codes and coding structure were discussed (EJ, MCØ), and codes clustering around similar concepts were merged into broader codes or code groups, leading to a second coding structure. Interviews were then recoded accordingly (MCØ). Fourth, we identified candidate themes (MCØ, EJ). Finally, the themes were reviewed and discussed in the whole research group (MCØ, EJ, TW, OR, EH). Extracts from interviews have been included as quotes to increase transparency and improve the reader’s insight into the analyses. These have been translated and linguistically revised for readability without altering the content. NVivo 12 (QSR International) was used in the coding process.

### Ethics

The study was approved by the Norwegian Center for Research Data (ref. no. 141435). All participants received written and verbal information about the study's main objective, i.e., ‘to elucidate the decision-making process leading to a decision to transfer or observe a patient with isolated head injuries.’ All signed a written consent to participate before the interview started. All participants had the right to withdraw from the study at any point during or after the interview, which no one did. During the transcript process, all participants were given pseudonyms, and the information required to map deidentified data with personal information was stored at a separate, encrypted location. Participant characteristics are reported in intervals and hospital identities are not given to preserve participant anonymity.

## Results

### Participant characteristics

We conducted four focus group interviews with a total of fifteen participants (Table 1). Their median age was 38 years (interquartile range [IQR] 30–44), 40% were female, and the median surgical experience was 4.5 years (IQR 2–13).

**Table 1:** Characteristics of study participants

Participant number	Hospital level	Age (yr)	Sex	Surgical speciality	Role	Surgical experience (yr)
1	Acute care trauma hospital	40–54	M	Gastrointestinal	Attending	11–15
2	Acute care trauma hospital	40–54	M	Orthopedic	Resident	1–5
3	Acute care trauma hospital	25–39	F	Orthopedic	Resident	1–5
4	Acute care trauma hospital	40–54	F	Gastrointestinal	Resident	6–10
5	Acute care trauma hospital	25–39	F	Gastrointestinal	Resident	1–5
6	Acute care trauma hospital	25–39	M	Orthopedic	Resident	1–5
7	Acute care trauma hospital	25–39	M	Gastrointestinal	Resident	1–5
8	Acute care trauma hospital	25–39	F	Gastrointestinal	Resident	1–5
9	Neurotrauma Center	40–54	M	Neurosurgery	Resident	6–10
10	Neurotrauma Center	25–39	M	Neurosurgery	Resident	1–5
11	Neurotrauma Center	40–54	M	Neurosurgery	Resident	11–15
12	Neurotrauma Center	40–54	M	Neurosurgery	Attending	11–15
13	Neurotrauma Center	40–54	M	Neurosurgery	Attending	16–20
14	Neurotrauma Center	40–54	F	Neurosurgery	Attending	11–15
15	Neurotrauma Center	25–39	F	Neurosurgery	Resident	1–5

### Overarching theme and main themes

Analysis of the focus group interviews gave insights into the setting of the decision-making process and identified several factors influencing the transfer decision. These were organised in one overarching theme transcending six interrelated main themes. The overarching theme, ‘The chance of a favourable outcome’, describes how participants were constantly considering how various factors may influence the chance of a favourable outcome. Participants pondered the likelihood of the patient surviving, achieving favourable rehabilitation outcomes, and a good post-injury quality of life. Many participants expressed statements like what Participant 8 (surgeon) said: *‘Can they withstand an operation, and if so, if one operates on them, what do you save them back to?’*.

**Fig. 2:**
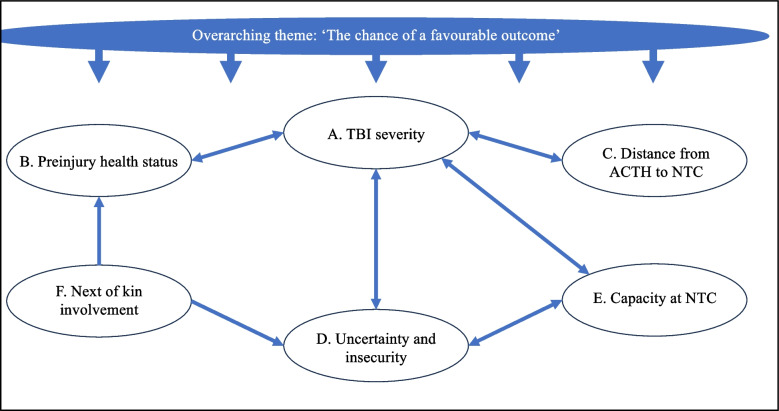
Code tree of the overarching theme and main themes. Arrows between themes reflect how they relate to and impact each other. Bi-directional arrows signal that the importance of one theme on the transfer decision can influence or be influenced by other themes; a single-directional arrow signals unidirectional influence from one theme on another. All themes are considered in light of the overarching theme

The main themes reflect how surgeons’ and neurosurgeons’ decision-making process was dynamic and multifaceted and how factors within themes were interrelated. They were: (A) ‘Establish TBI severity: Glasgow Coma Scale score and head CT’, (B) ‘Preinjury health status: comorbidity, functioning, and age’, (C) ‘Distance from ACTH to NTC: distance is time and time is brain’, (D) ‘Uncertainty and insecurity’, (E) ‘Capacity at NTC’, and (F) ‘Next of kin involvement’ (Fig. 2, Table 2). Figure 2 shows the interrelated nature between the overarching theme and main themes and Table 2 shows examples of empirical codes underlying themes.

**Table 2:**
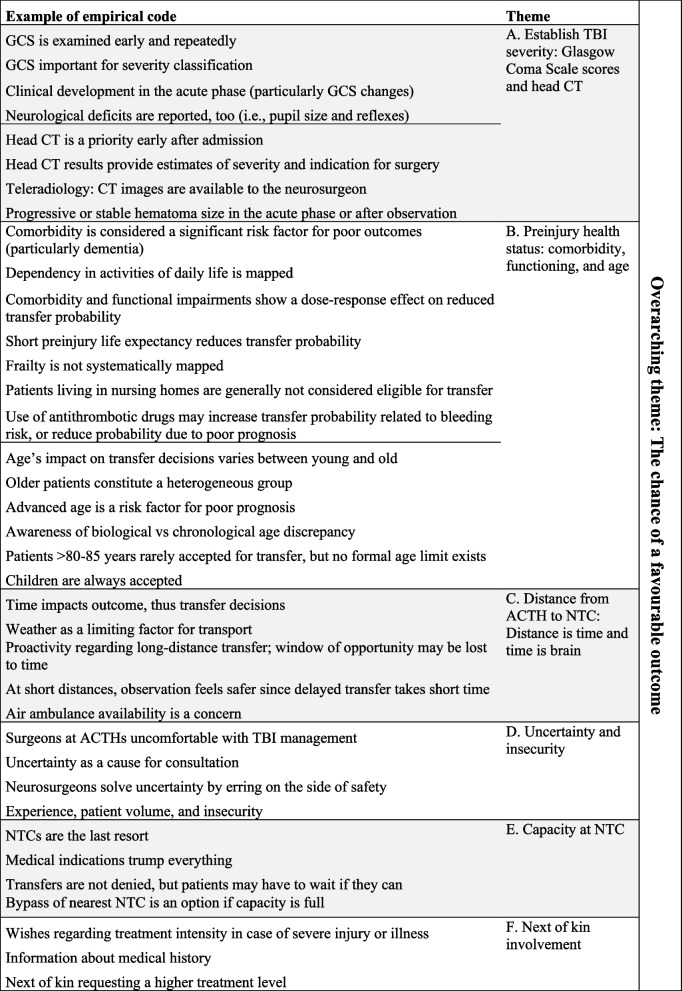
Overview of empirical codes and themes

### The setting of the decision-making process

The participants expressed that to reach a transfer decision, a telephone consultation was typically initiated by an orthopaedic or gastrointestinal surgery registrar following the initial clinical and imaging work-up and after a brief overview of medical history had been obtained. The reported objectives of the telephone consultations were to address any uncertainties related to the appropriate level of care, obtain backing for non-transfer decisions, seek guidance on follow-up plans for patients not being transferred, or provide neurosurgeons with information regarding patients requiring immediate transfer.

Both surgeons and neurosurgeons expressed that the threshold to initiate a telephone consultation was low and experienced that the accessibility of neurosurgeons was excellent:

*‘So, if I am uncertain at all, I talk to the neurosurgeons. […]. Whether it is 2 AM or 2 PM, they’ll do it. And they’ll discuss with their attending and all. So, I think they are good at being there for us.’* (Surgeon, participant no. 2).

Neurosurgeons described patients eligible for transfer as those whose injuries required specialised care exclusively provided at NTCs while not having characteristics that would impede the potential benefit of the transfer.

*‘It ultimately comes down to whether the injury is too mild or too severe. […]. Too mild reflecting that the probability that they’ll need surgery or neurointensive care is very, very small. And too severe; the prognosis is so poor that they won’t benefit from treatment.’* (Neurosurgeon, participant no. 14).

### Theme A: Establish severity: GCS scores and head CT

The participants stated that GCS scores and head CT imaging results were the starting points of all interhospital communication because they allowed for estimating TBI severity and identifying patients eligible for interventions according to guidelines. Information about trends over time were considered valuable, including lateralising neurological signs and GCS scores, and CT imaging was a priority early after admission. One surgeon stated that after a patient with head trauma came to the emergency department (ED), they were taken *‘right to the CT unless the GCS is so low that […] doing a CT has no point… to avoid any delays before transferring the patient.’* (Surgeon, participant no. 7).

The importance of joint teleradiological access to CT images was emphasised by both surgeons and neurosurgeons, facilitating expert advice on interpretation and subsequent treatment.

### Theme B: Preinjury health status: comorbidity, functioning, and age

Throughout all interviews, surgeons and neurosurgeons consistently emphasised the influence of preinjury health status on the transfer decision. These included i) comorbidities, ii) functional impairments, and iii) advanced age. Preinjury health status was assessed in close relation to the previous theme (A): if GCS and CT results indicated transfer to an NTC, the presence of comorbidity, functional impairments, or advanced age could lead to a non-transfer decision with a dose–response effect because of their association with poor outcomes.

*‘But what is often lacking is precisely that information [*regarding preinjury health status*] when they call with the results of a CT scan of the head and say that the patient has a GCS of 11. And then you wonder about that whole package of information […] about family, where they live, what medications they use, antithrombotics, and all that. It’s difficult to obtain in the acute phase.’* (Neurosurgeon, participant no. 14).

Comorbidities, functional impairments, and advanced age were considered interrelated, and participants expressed the importance of evaluating them together.

*‘These elderly – it’s not that they are not accepted solely because of their age (…), but rather […] the question is often: “Is this someone who should potentially undergo surgery now or in case they are deteriorating?”. And in many cases, the answer is no, primarily due to considerations of comorbidity and functional status. One considers the chances of a positive outcome are very slim and as a result, it becomes more of, like… an ethical point.’* (Neurosurgeon, participant no. 10).

#### The role of comorbidities

Comorbidities were mapped broadly to get information about the number and nature of patients’ diseases. The emphasis was not on specific diagnoses but rather on understanding the overall burden of risk factors that could contribute to poorer outcomes. The exception was advanced dementia and advanced cancers, which were frequently referred to as a contraindication for transfer, given their significant impact on a patient’s overall condition, rehabilitation potential, and life expectancy. Antithrombotic drugs were explicitly mentioned as a factor related to comorbidity. Their associated risk of haemorrhage expansion could lower the threshold for transfer in cases where proactivity was warranted or lead to rejections where they had contributed to a too-large hematoma.

#### The role of functional impairments

Assessments of functional impairments focused on functioning in activities of daily life and whether the patient received home or institutional nursing care. Residency in nursing homes was emphasised as an important marker of significant functional impairments. As one surgeon stated: *‘It’s the level of functioning, isn’t it? The cognitive state before the injury; is he active, lives at home, uses some aids – if he’s functioning, really’* (Surgeon, participant no. 5)*.* Another said: ‘*However, even among those who live at home, there is a wide range, making it challenging to assess their condition accurately over the phone without having seen the patient’* (Neurosurgeon, participant no, 10)*.* Scoring tools, such as frailty scales, were not systematically used.

#### The role of age

The impact of age on transfer decisions varied depending on whether the patients were young or old. The heterogeneity within the older population was emphasised, making the participants cautious of denying patients transfer based on age alone. As one surgeon noted (participant no. 7): *‘Many older people are very fit these days.’.* The participants also highlighted the discrepancies between biological and chronological age.

When asked about when age became an independent factor in considering non-transfer, one participant responded:

‘*A patient can be 89 years old and still be able to drive a car, and he can be 67 years old and not have a chance to walk across the floor. So, it is not possible to answer your question.’* (Surgeon, participant no. 2).

However, other participants acknowledged that advanced age could independently contribute as a risk factor for poor prognosis and, thus, non-transfer. Although specific age limits for transfer to NTCs were not used, there seemed to be a threshold around ages 80–85 years where the willingness to accept transfers significantly decreased.

*‘Once patients reach the age range of 80 to 85, we begin to factor their age into the decision-making process. However, it’s crucial to exercise caution and not solely rely on age as the determining factor, as there is significant variability within this age group.’* (Neurosurgeon, participant no. 13).

For younger patients, age had limited influence on the transfer decision. As comorbidities and functional impairments are usually less prevalent in younger age groups, the primary factors guiding the decision were the GCS scores and CT imaging results. Neurosurgical participants unanimously expressed a low threshold for accepting children for transfer. Despite significant initial findings, the chance of a positive outcome allowed younger patients to remain eligible for transfer.

### Theme C: Distance from ACTH to NTC: distance is time and time is brain

Participating neurosurgeons stated that information about time from injury was important for two reasons. First, because time that had passed allowed assessment of clinical development, in which deterioration could increase transfer indication and vice versa. Second, because a long time from injury to treatment could impact the chance of good outcomes because of secondary insults. Long transfer distances and transport difficulties challenged timely care due to bad weather or air ambulance availability, and long transfer distances could directly lead to a non-transfer decision because the window of opportunity would be lost to time.

*’ If the patient had been here in the first place, then it would have worked, but if there are five hours of transport time, then it won’t work.’* (Neurosurgeon, participant no. 12).

For patients with a borderline indication for transfer, neurosurgeons described confidence in observation at the ACTH if the transfer time was so short that they could swiftly reach the NTC should they deteriorate. For patients with a longer transfer distance, neurosurgeons stated a low threshold to accept patients for transfer in the first place because *if* they deteriorated, they would not reach interventions in time. There were, however, conflicting views on this practice from participants who had worked at hospitals located both near and remote from NTCs, who had experienced a higher threshold to transfer such patients when the transfer distance was long:

*‘And then you have those who are somewhat uncertain, who are relatively healthy, alert, not too old, so they are mostly transferred for observation just to be on the safe side. Especially if they are far away.’* (Neurosurgeon, participant no. 10).

### Theme D: Uncertainty and insecurity

Uncertainty about appropriate clinical management was a frequent cause of initiating a telephone consultation for surgeons at ACTHs. They stated high confidence in neurosurgeons’ judgment to identify eligible patients, and disagreement about the transfer decision was rarely experienced.

*‘There have been instances where I’ve made the decision to transfer a patient, and then I’ve been told […] that “It’s not necessary after all”. […] Occasionally, it is hard to fully understand why. But […] it is a speciality of its own, so they have a deeper understanding. I mean, they are used to seeing the long-term consequences of various conditions, which I do not have a complete overview of.’* (Surgeon, participant no. 4).

Neurosurgeons expressed that if there was remaining doubt after all relevant information was gathered, particularly regarding factors captured by Themes A and B, they tried to eliminate uncertainty when making decisions by choosing to err on the side of transfer. Both focus groups with neurosurgeons strongly expressed statements such as *‘It is never wrong to accept a patient for transfer’* and *‘If you’re in doubt, you’re not in doubt, then you accept the patient for transfer’*. They expressed that they rather accepted a patient too much than too few and that the department culture supported such a practice. They mentioned discussions with colleagues while on-call and plenary meetings in the department to discuss decision-making processes and adjust the threshold of transfer decisions.

Insecurity about having to take responsibility for TBI patients at ACTHs was mentioned as a factor that could influence transfer decisions. Patients who could be observed at the ACTH according to the neurosurgeon’s judgment, but where the surgeon at the ACTH opposed this because of insecurity, could undergo transfer to the NTC after some discussions and pending on capacity. Neurosurgeons considered this in light of varying competence at hospitals with different patient volumes and had higher expectations of larger than smaller ACTHs.

### Theme E: Capacity at NTC

Neurosurgeons considered that NTCs’ ward and ICU capacity had only a minor impact on transfer decisions. Surgeons at ACTHs stated that they had never experienced rejections because of capacity. However, full wards or ICUs were not an infrequent issue. Still, if transfer to an NTC was medically indicated, the neurosurgeons saw their role as a last resort for patients who needed their services. They either accepted transfer and reorganised their in-hospital resources or facilitated bypass to a neighboring region’s NTC.

*‘If there is a head injury that makes the patient somehow belong here… I have never experienced that it must somehow be changed. Then one must rather move something else. I have never experienced someone saying: “Yes, that patient should have been here, but unfortunately, it is not possible now”.’* (Neurosurgeon, participant no. 11).

Full capacity could, however, lead to a non-transfer decision regarding patients with a borderline indication or where they otherwise could have accepted because of insecurity at the ACTH (related to Themes A and D).

*‘With these kinds of difficult decisions, capacity is something that contributes to possibly holding them back rather than accepting them, especially if our monitoring and intensive care units are already at full capacity. The intensive care and observation or intermediate units are often very crowded here at the hospital.’* (Neurosurgeon, participant no. 13).

### Theme F: Next of kin involvement

Participants stated that next of kin involvement served an important role when the severity of the situation indicated interventions (Theme A) but more detailed information about preinjury health status or expressed wishes about treatment limitations in severe situations was necessary to make the final decision (Theme B).

*‘Yes, neurosurgeons often want to know if we have spoken to the relatives. So we often do that. […]. They usually ask, “What do the relatives want?”.* (Surgeon, participant no. 5).

Next of kin involvement was also experienced when patients were declined for transfer from the ACTH to the NTC because of unsalvageable injuries or because observation was deemed sufficient. Then, neurosurgeons frequently experienced repeated telephone consultations with the surgeon at the ACTH who was under pressure to do more. In some cases, pending in part on capacity (Theme 5), patients were accepted.

## Discussion

In this study, we explored the decision-making process for interhospital transfer of patients with isolated TBI and elucidated the factors influencing these decisions to increase the understanding of access to NTC care for older adults. Under the overarching theme ‘The chance of a favourable outcome’, six main themes were identified, which captured clinical and system-level factors that influenced the decision-making process in an interrelated way: (A) ‘Establish TBI severity: Glasgow Coma Scale score and head CT’, (B) ‘Preinjury health status: comorbidity, functioning, and age’, (C) ‘Distance from ACTH to NTC: distance is time and time is brain’, (D) ‘Uncertainty and insecurity’, (E) ‘Capacity at NTC’, and (F) ‘Next of kin involvement’. These findings suggest that on-call surgeons and neurosurgeons responsible for making transfer decisions for TBI patients apply a broad approach to gathering relevant information to make patient-centred decisions.

The surgeons and neurosurgeons applied a broad approach to making transfer decisions where more factors than TBI severity were involved (Fig. 2). Their statements expressed a gatekeeper function that aimed to identify patients who could benefit from specialised neurotrauma services at NTCs (Overarching theme) while protecting service capacity. Themes A-C reflect risk factors for poor outcomes in the literature; secondary insults worsen prognosis [23], and comorbidities, functional impairments, and advanced age have been associated with lower survival rates, poorer functional outcomes, and limited access to rehabilitation following TBI [14, 15, 24]. Themes D-F reflect factors with a ‘softer’ influence on the decision, comparable to an interview study of 27 trauma surgeons and nurses in the USA who reported that clinician experience and system-level capacity influenced transfers in a general trauma population [25]. Advanced age, as identified as a significant clinical feature by participants in this study, has consistently been associated with reduced transfer rates in retrospective studies from general trauma and TBI populations [5, 8, 9]. This study substantially adds to the knowledge about transfer-influencing factors, particularly regarding how preinjury health status is evaluated and weighted and the factors captured by themes D-F.

The broad approach to decision-making identified in this study aligns with the established criteria for making healthcare priorities in Norway; benefit, resource, and severity [26]. An anticipated benefit from NTC care was decisive for participants to conduct transfers (Overarching theme). Transfer and NTC care are resource-demanding, so if the benefit were deemed marginal, such as from a too-low or too-high injury severity, significantly impaired preinjury health status, or too-long transfer time, transfer would be declined. Furthermore, the approach aligns with the medical ethical principles of beneficence and non-maleficence, reflecting that decisions should also not lead to harm, e.g., by unnecessary transfers associated with risks [27]. The alignment with these principles is not unexpected, as they are fundamental in training and applied in everyday elective decision-making. However, the approach is much more nuanced and complex than trauma guidelines’ one-sided focus on identifying patients eligible for transfer [10–12], thus leaving it to the clinical environment to define non-eligible patients.

The commitment of participants to consider patients’ overall health status rather than solely relying on age must be considered a strength of the current decision-making process. All four focus groups with surgeons and neurosurgeons emphasised this approach, indicating a shared understanding of the relevant factors influencing transfer decisions. Notably, only comorbidities, functional impairments, and advanced age were mentioned as influential, despite a focus during interviews to ask open-ended questions about what they regarded as important when making transfer decisions. Other factors, such as injury mechanisms, were not emphasised, and notably, sex, ethnicity, and drug misuse were not reported to influence decisions. These findings converged with our group's recent population-based observational study, which revealed that sex and injury mechanisms were not significantly associated with interhospital transfer from an ACTH to an NTC when adjusted for other factors [4].

The multidimensional approach described by the participants to assess patients’ diseases, functional status, and age aligns with the concept of frailty [28]. Interestingly, the term “frailty” was not explicitly mentioned by the participants. Galimberti et al. recently showed an association between frailty and six-month functional outcomes following TBI and recommended incorporating frailty screening during the initial investigation of TBI patients [29]. The present study shows that clinicians already apply comparable thinking, although without using validated tools. Thus, the screening tool Clinical Frailty Scale (CFS), which has been shown to correlate well with clinically meaningful endpoints in trauma and ICU populations and is feasible to collect in an ED setting, warrants future investigation in a TBI setting [30–35].

The various factors that influenced the decision-making process relied on access to accurate patient information and handover and interpretation of that patient information. Errors at any of these stages could result in misjudged transfer decisions. For instance, there could be inaccuracies in the information obtained from electronic patient records and the interpretation of patient or next of kin information regarding comorbidities and functional levels. Furthermore, the expression of this information during the telephone consultation could be over- or underestimated by both parties. Misjudged transfer decisions could lead to erroneous acceptance and denial of transfers, which both carry potential patient risks. Notably, neurosurgeons expressed a clear awareness of the risks of wrongful transfer rejections and stated their intention to prioritise patient safety by erring on the side of caution.

## Strengths and limitations

First, a larger sample size would have strengthened the study by possibly bringing forward more factors or differing viewpoints on the already identified factors. However, there was strong coherence about the clinical factors affecting transfer decisions in the focus group interviews. Second, ensuring truly unfiltered statements from participants is challenging, a potential disadvantage of focus groups. For example, age’s role in treatment-limiting decisions may be controversial. Assuming that most participants would avoid being seen as ageists in front of colleagues, the reported impact of age may be faded down, thus being reported as solely secondary to comorbidity and functional impairments until the advanced age of 80–85 years. However, it converges with findings from quantitative data analysis on the same patient population, increasing the findings' reliability [4]. Third, we confined recruitment to a Norwegian hospital setting which may have reduced the international transferability of findings. Nonetheless, many features were present that are common denominators in many trauma systems internationally, including centralised neurosurgical facilities necessitating interhospital transfer, bypass criteria of local hospitals, implementation of international TBI guidelines, and ageing populations. Furthermore, the participant background, setting, and patient cases would likely be similar at other hospitals, yielding some transferability. We successfully recruited a purposeful sample that reflected hospitals with various sizes and distances to NTCs and participants with real-world relevant decision-making experience representing both sexes and various specialities and years of experience. All interviews were conducted without the presence of any heads of departments, at locations where the participants work and are well-known, during regular working hours, which is likely to have contributed to a safe atmosphere and an honest discussion.

## Conclusions

Through analysis of qualitative interviews, we identified several clinical and system-level factors surgeons and neurosurgeons consider when making decisions about interhospital transfer for patients with isolated TBI. They emphasise individual patients’ risk factors and overall health status and aim to make patient-centred decisions guided by the chance of achieving a favourable outcome. This increases our understanding of access to NTC care for patients admitted to non-neurosurgical hospitals.

## Supplementary Information

Supplementary Material 1 : COREQ checklist. Norwegian criteria for field triage and transport destination of injured patients. Interview guide.

## Abbreviations

ACTH: Acute care trauma hospital
CT: Computed tomography
GCS: Glasgow Coma Scale
ICU: Intensive care unit
NTC: Neurotrauma Center
TBI: Traumatic Brain Injury
