# Supplementary material for: Decision-making in interhospital transfer of traumatic brain injury patients: exploring the perspectives of surgeons at general hospitals and neurosurgeons at neurotrauma centres
Source: BMC Health Serv Res. 2025 Feb 11;25:234. doi: 10.1186/s12913-024-11968-z (PMC11817790; doi:10.1186/s12913-024-11968-z)
Supplement: Supplementary file 1 — Supplementary Material 1 : COREQ checklist. Norwegian criteria for field triage and transport destination of injured patients. Interview guide. [file 12913_2024_11968_MOESM1_ESM.docx]

**Supplemental Online Content**

To *Decision-making in interhospital transfer of older traumatic brain injury patients: exploring the perspectives of surgeons at general hospitals and neurosurgeons at neurotrauma centres*

List of contents:

- Appendix 1: COREQ checklist
- Appendix 2: Field triage and bypass criteria
- Appendix 3: Interview guide

**Appendix 1: COREQ criteria**


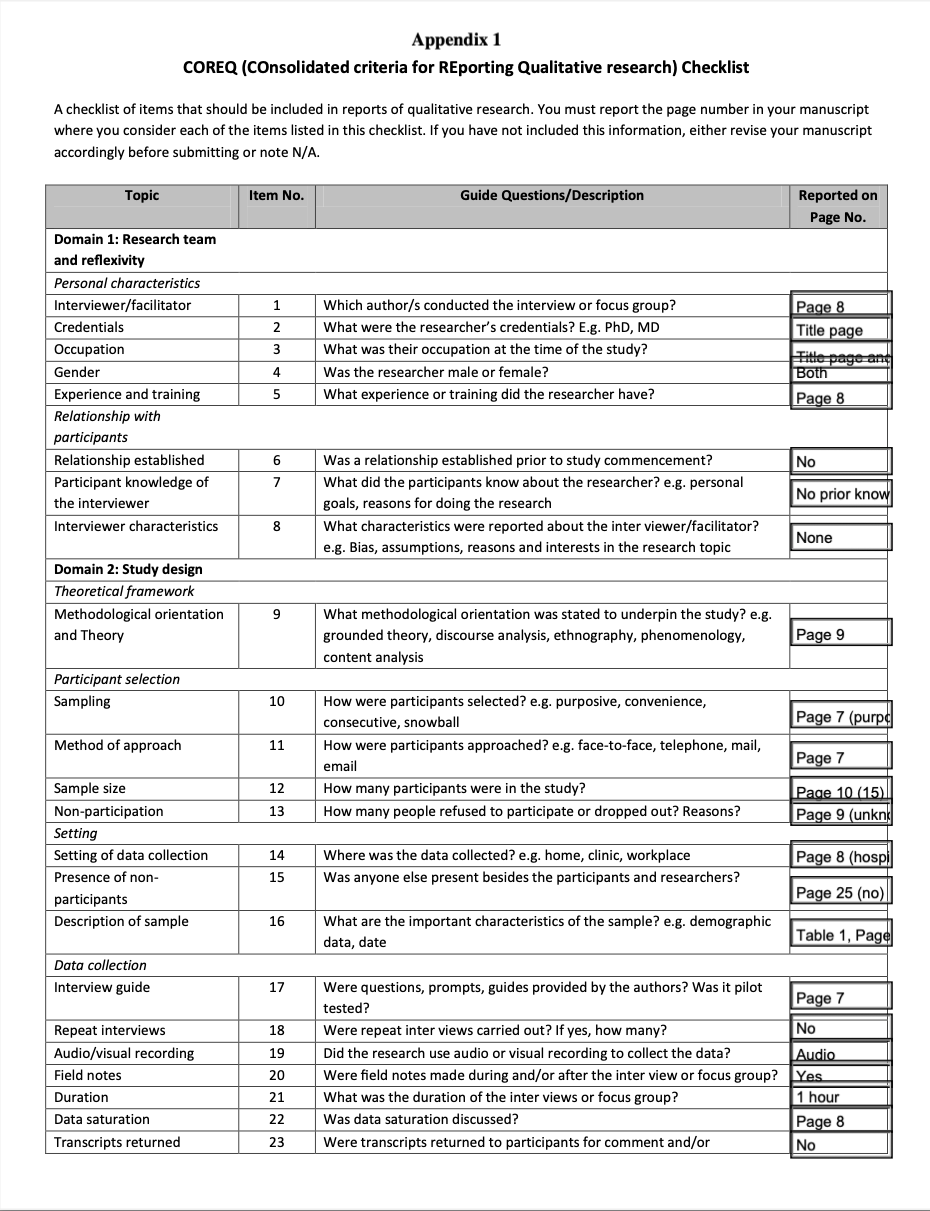


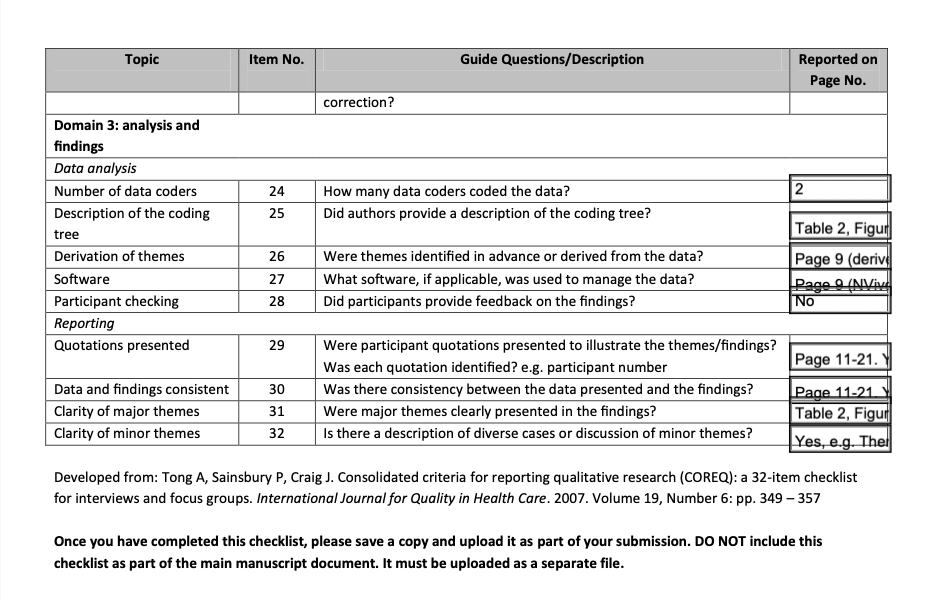


**Appendix 2: Field triage and bypass criteria.**


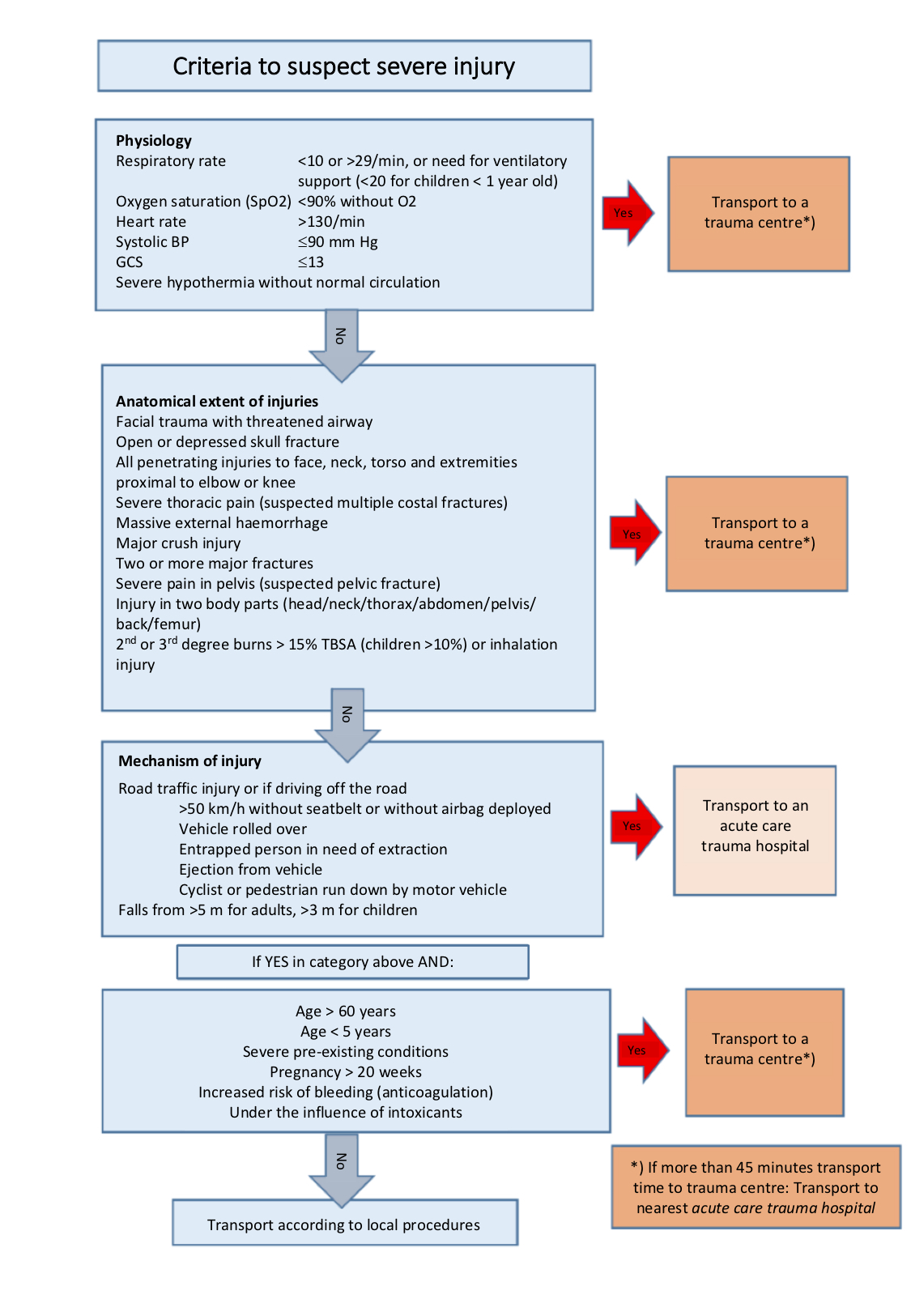


**Appendix 3: Interview guide**

*Two versions of the interview guide were developed with minor alterations in wording to ensure applicability to both professional groups. The sequence of topics was open and followed what was mentioned by participants.*

Opening question: Mapping how this process takes place.

Opening statement to neurosurgeons: *Imagine that you are on call and receive a consultation about a patient who has suffered a head injury and has been admitted to a local acute care hospital with trauma services. Describe how this consultation takes place.*

Opening statement to surgeons: *Imagine that you are on call and you have received a patient at the ED in your hospital. After the initial investigation, you find that his/her main problem is a head injury. Please describe how you go ahead to evaluate and consult with anyone regarding the need for referral to a higher treatment level.*

• Who makes the call? Who do they call? Is it you, the trauma team leader, or others?

• What type of patients are they calling about?

• What information do you ask for?

• How accessible are you, and do you often face conflicts in availability?

Making a decision

• Who decides whether a patient should be transferred or not?

• What are the decisions based on?

• Do you review the images together?

• What resources do you rely on when making a decision? (e.g., guidelines, trauma plan, on-call physician, textbooks, studies, etc.)

• For younger and older patients, are pediatricians or geriatricians involved?

Uncertainty and disagreement

• Are you ever unsure about what the right decision is?

•What causes this uncertainty?

• How does it feel when you are unsure about whether it is right to accept the patient for transfer or not?

• Have you ever disagreed with the person who calls in and wants to transfer a patient?

• How does it feel to disagree with the person consulting you?

• Can you provide specific examples of this?

When the patient is not accepted for transfer

• In what situations do you choose not to accept the patient for transfer?

• Why would you sometimes choose to say no to patients that the local hospital has assessed as needing transfer? Are there any non-clinical factors at play?

• What happens next to patients who are not transferred? Another CT scan? Another consultation? Plan?

• What are your thoughts on the care provided to head injury patients at local hospitals?

Capacity and resources in the department

• Tell us about the capacity in your department and whether it is something you have to consider during your shift.

• How does it affect the consultation conversations? What does it do to the prioritization?

*I want to talk more about what you said about (pick up on any of*

Comorbidity

• What role does comorbidity play in the transfer evaluations?

• Explain the assessments you make when it is reported that the patient has pre-existing conditions and the considerations you have regarding transfer.

• What do you understand by the term "comorbidity"? Do you differentiate between diseases and activities of daily living (ADL)?

• Which diseases carry the most weight in the assessments?

Age

• Address anything that has been mentioned about age so far and encourage further discussion on this topic.

• How does age come into play when you assess these consultation conversations?

• How heavily is age weighed compared to other factors?

• From what age does age itself begin to influence:

- the patient being considered old?
- age being seen as an independent risk factor?
- decisions regarding interventions?

• Why does age impact:

- urgency?
- interventions/procedures?
- level of treatment?
- transportation or other aspects we have discussed?
- Why does age matter?

Characteristics of older head injury patients

• How do you experience treating older patients with head injuries? If this has been discussed: What makes older patients a challenging group?

Feedback from colleagues

• Describe the discussions/questions during the morning meeting after receiving an older patient from another hospital during your shift. What if the department is full? What if no surgical treatment is given and the patient is sent back the same day?

• Is it different if the patient is young?

Prognosis

• What is your perception of the outcomes for older patients with head injuries compared to younger patients?

• Are there any aspects we have discussed now that you highlight in cases where treatment limitations without transfer are decided?

Patient's wishes and the role of family members

• The need for information can be significant. Do you find that patients or family members are involved in this process?

• How would you describe the role of family members in the assessment of whether a patient should be transferred or not? (Advocates?)

• What about the patient's wishes?

Conclusion: Do you have anything else on your mind? Is there anything you would like to add or any relevant aspects that I haven't asked you about?
